# Evaluation of Commercial Probes for On-Line Electrical Conductivity Measurements during Goat Gland Milking Process

**DOI:** 10.3390/s120404493

**Published:** 2012-04-10

**Authors:** Gema Romero, Jose Ramon Díaz, Jose Maria Sabater, Carlos Perez

**Affiliations:** Department of Tecnología Agroalimentaria, Escuela Politécnica Superior de Orihuela and Department of Ingeniería de Sistemas y Automatica, nBio Group, Quorum V Building, Campus de Elche, Universidad Miguel Hernández, Spain; E-Mails: gemaromero@umh.es (G.R.); jr.diaz@umh.es (J.R.D.); carlos.perez@umh.es (C.P.)

**Keywords:** electrical conductivity, on-line measurements, goat milk, mastitis detection

## Abstract

The measurement of the milk electrical conductivity (EC) during mechanical milking has been widely studied for mastitis detection on cows because its improving of welfare and animal health, although research about small ruminants is scarce. The aim of this study was to evaluate the performance of three commercial conductimeters to be used during mechanical milking of small ruminant halves, especially Murciano-Granadina goats. The objective of this research was to integrate the probes on the milking unit and to check the suitability of the probe selected. The results presented in this research have guided authors to discard the commercial probes and to establish the requirements of a new probe design that is briefly outlined in the conclusions of this contribution.

## 1. Introduction

Food Hygiene regulations approved in 2004 and 2005 by the UE aim to assure public health protection, as well as animal health and welfare. Good agricultural and farming practices need to be implemented and promoted in the primary sector as a first step for a future analysis implementation of hazard and critical control points (HCCP).

Electrical conductivity (EC) devices during milking may improve mastitis detection and thus improve small ruminant welfare and health. It has been widely studied for mastitis detection on cows, due to the capability of automatization in the milking machine [1–4]. The main advantage is the results can be achieved on-line during milking, with objective measurements and with a relatively lower cost, if compared to other mastitis detection methods (bacteriological analysis, SCC, serological, California Mastitis Test, etc.).

**Figure 1.**
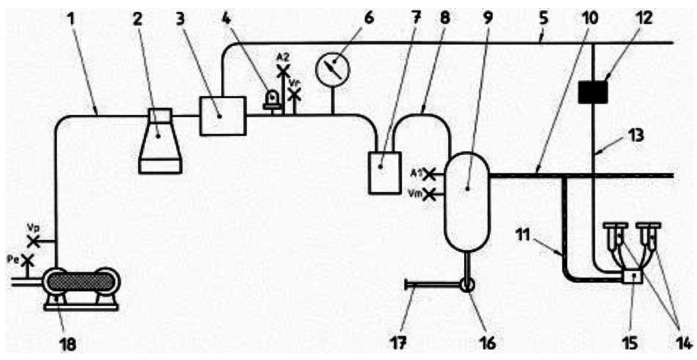
Example of a pipeline milking machine. (extracted from ISO 3819).

Several publications around the automatic measurement of EC for mastitis detection on cows can be found on specialized literature [5–7]. The different methods for on-line EC measurement differ if the measurement is done for the milk coming from the whole udder or at every gland level. In the first case, probe sensors are allocated in the long milk tube (11 on Figure 1) that is the tube beyond the claw (15 on Figure 1), and in the second case in the short milk tube (tubes between 14 and 15 on Figure 1). In both cases EC data are logged during the milking time, when the milk is following through the tubes. Data is processed on a central computer that uses a model or algorithm to detect if the udder or the gland is candidate to be affected by mastitis.

Nevertheless, literature about the use of EC measurement for mastitis detection of small ruminants like caprine is very scarce [8–10]. Ying [10] published that mastitis in Saanen breed produces an increase of 0.2 mS/cm (from 5.6 to 5.8 mS/cm) while in Alpina breed the effect was a higher decrease of EC (from 6.1 to 5.4 mS/cm). This previous work invited us to study the different factors related to EC and the association of intramammary infection with EC in the local breed of our region (Murciano-Granadina) [11] obtaining different increases depending on the pathogen affecting. Other aspect was researching on automation of EC measurements during milking by gland, instead of by animal, for mastitis detection of small ruminants.

The aim of this study was to evaluate the performance of three commercial EC probes for their use by gland during small ruminants milking, and to establish the requirements of the desired probe. The goal of this research is to integrate the probes on the milking unit and to check the suitability of the probe selected. The results presented in this research have guided authors to study a new probe design that is briefly outlined on the conclusions of this contribution.

## 2. Materials and Methods

All the experiments were carried out in the Universidad Miguel Hernández de Elche(UMH, Spain), with the participation of Animal Production and Systems Engineering areas. First experiment was carried out at the laboratory of Systems Engineering, the second one was carried at the laboratory of Animal Production area and the third one was carried out at milking parlour level in the educational and experimental farm of small ruminants of the UMH. Materials and methods of each experiment are detailed below.

### 2.1. Objectives

Prior to trying to develop a suitable system for on-line EC measurement during goat milking, it is necessary to analyze the requirements of the EC probes to be inserted in an on-line system where the intermittent vacuum needed for the massage and the flowing air introduces noise on the measures. Finally, we need to evaluate the performance of commercial probes to suit these requirements.

That is, the objectives covered on this paper are, firstly to study the special requirements of an on-line EC measurement system during milking, specially the effect of fat, vacuum and cleaning process on the temporary performance of the probes, and in second term to evaluate the performance of different commercial equipments for EC measurements, with different physics (inductive, conductive) and geometries in order to know the most desirable properties.

All the tested equipments were acquired to the special tasks of measuring the electrical conductivity of fresh goat milk. The expected result was to be able to define the most suitable physics and geometry of probes for the on-line EC measurement during milking.

### 2.2. Experiments

The study was divided in three experiments, two of them were carried out at laboratory level: off-line experiment and on-line testbed experiment, meanwhile the third experiment was carried out on a real milking parlour, in order to check the performance of the system at field conditions. Depending on the former objectives, next experiments were made in the laboratories of the group.

#### Off-line measurement

The off-line experiment had the aim to select conductimeters with proper measurement to goat milk EC range and to design a prototype to be incorporated to the milking machine in order to take on-line measurements. The measures were done on static process, so the dynamics of the system did not introduce noise.

#### On-line testbed

On this experiment, milking conditions were artificially simulated and a first evaluation of the on-line performance of the commercial probes were done. The geometry of the probes and short milk tubes were studied and a plastic box to accumulate the milk was designed for each probe. The variation on time and cleaning process of the probes were evaluate.

#### On-line milking parlour

After the second experiment, the selected commercial probes were tested in on-line conditions, at the milking parlour available on the Universidad Miguel Hernandez, during the lactation period of 24 goats. This experiment tested the system on a real milking parlour.

## 2.3. Materials

**Figure 2.**
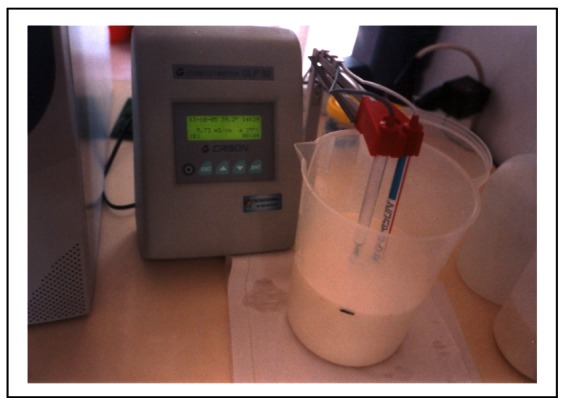
Reference conductimeter. Laboratory EC probe.

**Figure 3.**
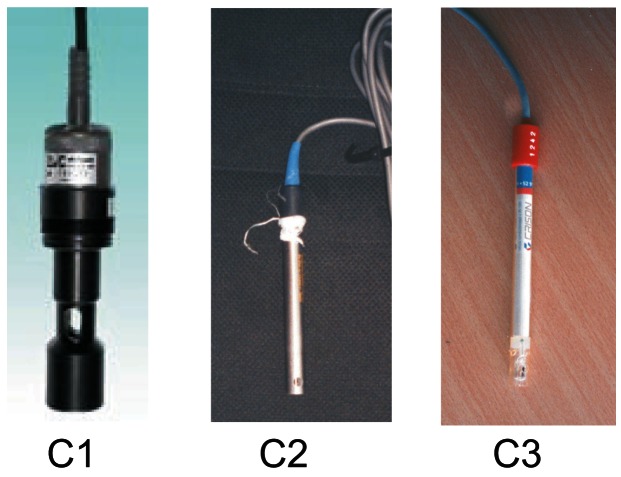
Commercial probes used on experiments.

**Figure 4.**
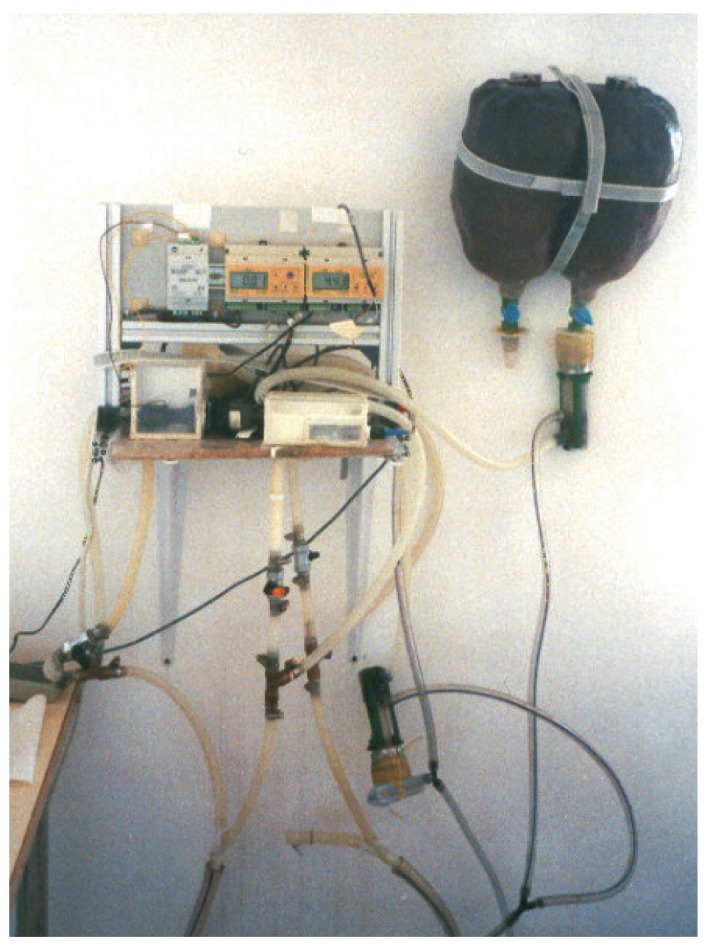
On-line testbed for evaluation of probes.

Next materials were used during experiments:

- Fresh goat milk extracted from Murciano-Granadina goats in a commercial farm.
- UHT cow milk acquired on a supermarket.
- Several saline solutions (NaCl) with different concentrations (different EC).
- Water bath, with regulation of temperature.
- Laboratory EC conductimeter (GLP32 model, Crison Instruments, Allela, Spain), with automatic temperature correction (25 °C or 20 °C). The probe uses conductivity to measure EC, it has two parallel platinum planes (52–92 model from Crison Instruments, Allela, Spain) and a PT1000 temperature probe (model CAT Pt1000 from Crison Instruments, Alella, Spain). This probe was taken as gold reference to check the measures of the other probes and it was calibrated with standard patrons (1,413 S/cm and 12.88 S/cm) every day (See Figure 2).

- Three conductivity meters that were used on the experiments are shown on Figure 3, meanwhile dimensions are summarized on Table 1.
  - Conductivity meter C1. Model C3655 from B&C Electronics Srl, (Carnate, Milan, Italy), with an inductive probe covered by PVC (Model SI 315 from B&C Electronics Srl, Carnate, Milan, Italy), and an integrated temperature probe PT100. Automatic compensation of temperature is done at 20 °C with a compensation coefficient of 2%/°C. Range of measurement is 0–20 mS/cm. (See Figure 3).
  - Conductivity meter C2. Model C3630 from B&C Electronics Srl, (Carnate, Milan, Italy), with an inductive probe made of inox steel (Model 2731312 from B&C Electronics Srl, Carnate, Milan, Italy), and an integrated temperature probe PT100. Automatic compensation of temperature is done at 20 °C with a compensation coefficient of 2%/°C. Range of measurement is 0–20 mS/cm.
  - Conductivity meter C3. Model 524 from Crison Instruments, (Allela, Spain), with a conductivity probe made of platinum (Model 52–90 from Crison Instruments, Allela, Spain) and an integrated temperature probe PT100. Automatic compensation of temperature is done at 25 °C with a compensation coefficient of 2%/°C. Range of measurement is 0–199.9 mS/cm.
- Portable milking machine (Flaco©, Spain) for goats. This machine was used on the on-line testbed experiment. Milking parameters employed were 40 kPa of vacuum level, 90 puls/min of pulsation rate and 60% of pulsation ratio, usually employed for dairy goats.
- Artificial udder made of two halves with a global capacity of 2 liters per gland (see upper right part of Figure 4). A manual valve to regulate the milk flow was allocated on the lower part of each gland. After each valve, a plastic part with nipple shape allows to connect it with the milking machine. This artificial udder was used on the on-line testbed experiment.

**Figure 5.**
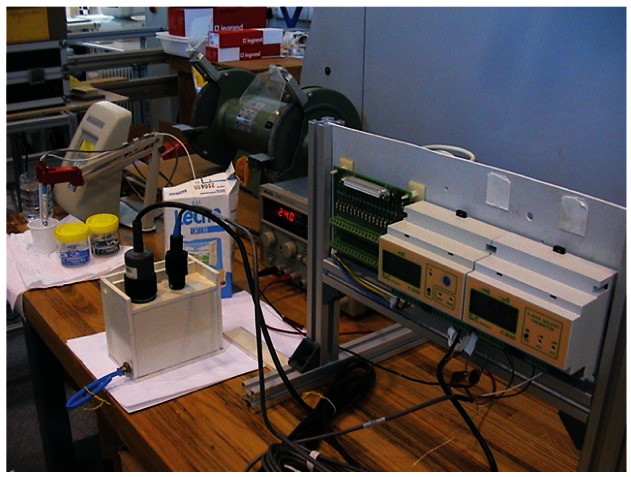
Off-line testbed for evaluation of probes. At rear is the laboratory EC probe and in front are C1 and C2 probes inserted at one measurement chamber.

**Figure 6.**
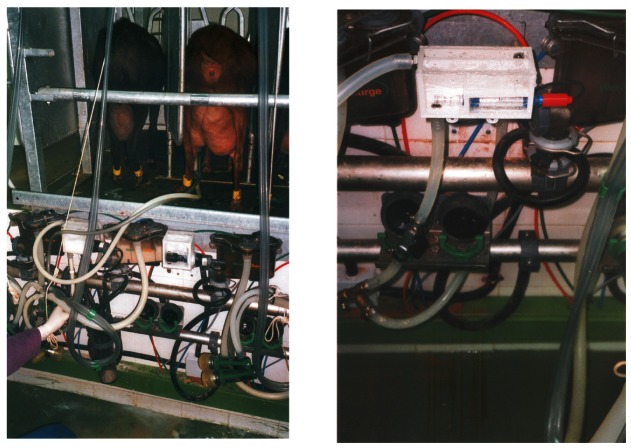
Detail of the probes in the milking parlour.

**Figure 7.**
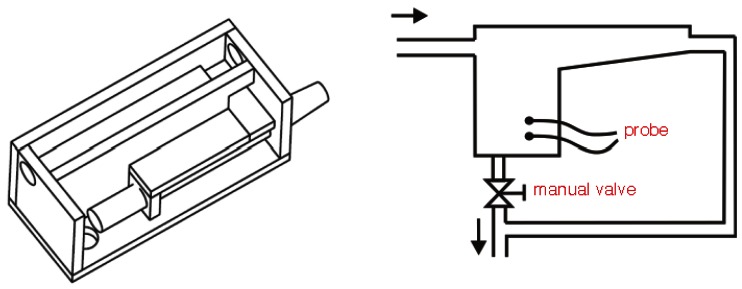
CAD drawing of one of the boxes. Scheme of the inlet and outlet ports.

**Figure 8.**
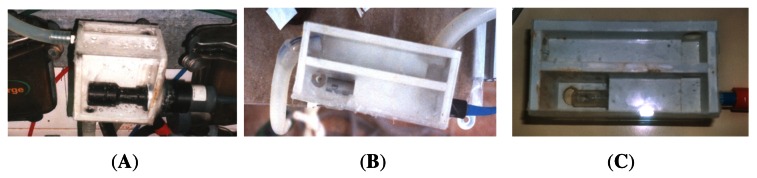
Hand-made boxes for C1 (**A**), C2 (**B**) and C3 (**C**) probes.

Each one of the probes were inserted on plastic hand-made chambers designed for each probe (see Figures 4, 5 and 6). These boxes have one input port for the milk and two outports, one for the vacuum and one more for the milk (see Figures 7 and 8). This last port has a manual valve that allows to fill the measurement chamber with milk and then, when the valve is opened, milk is evacuated and the measurement process starts again. The sensor probe is allocated on the measurement chamber, and the vacuum flow goes from one of the outports to the input port freely. Dimensions of the boxes varied depending on the dimension of the inner probe. Table 1 reflects the minimum dimension of the measurement chamber that was needed in order to allow the probe to be submerged enough to have a right measurement. These chambers and their dimensions were designed by simple static experiments submerging the probes manually during the off-line experiment.

Features that were taken account to select the conductivity meters were the range of measurement, the resolution, the temperature compensation and the dynamics of the measure.

- Resolution of the conductivity meter is the minimal unit of EC that can be read; higher the resolution is, lower the value of the minimal unit of EC is. Besides, the resolution depends on the range of measurement, it can be configured depending on the range. Resolution of laboratory probes should be around 1%–*2*% of the range scale, the standard deviation also being between 1% and 2%.
- Temperature compensation is the ability to modify the measure depending on the temperature of the solution. Temperature variations make change the EC due to the kinetics of fluid particles. Systems with temperature compensation offers the lecture of the EC as it was at normal temperature (usually 20 °C), despite of the real temperature. This compensation is expressed as a percentage, usually 2%/°C for ionic solutions and around 1.5%/°C for acids, alkaline solutions or high concentration solutions.
- Dynamics of the sensor is also an important feature. Some sensor can provide the measure in milliseconds but other uses more time. This is specially important for the temperature probes, as in our experiments, probes are inserted on variable conditions of temperature, from the milk temperature to vacuum conditions.

Other features considered for the probes and conductimeters selection were their functioning principles. Looking at the kind of conductimeters, we can find:

- Conductive probes, which are made of metallic electrodes (platinum or other metal), usually with ring shapes, and work measuring the resistivity of the solution to the electricity transfer from the positive to the negative electrodes. Electrodes are mounted over glass or PVC to avoid deformations.
- Inductive probes, which have the advantage that the fluid does not wet the electrical parts of the probe. Here, two inductively-coupled coils are used. One is the driving coil that produces a magnetic field and it is supplied with accurately-known voltage. The other forms a secondary coil of a transformer. The liquid passing through a channel in the sensor forms one turn in the secondary winding of the transformer. The induced current is the output of the sensor.
- Sanitary probes are inductive probes that have been built using sanitary materials that are not transformed during measurements and do not induces changes on fluid composition.

All the boxes used during experiments (see Figure 8) were hand-made built of PVC and transparent methacrylate for one of the walls so that the user can see the level of the milk on the measurement chamber and manage the manual valve in a proper way.

### 2.4. Methodology

#### 2.4.1. Off-Line Measurement

Figure 5 shows the setup for the off-line measurement of the EC on goat milk. Several boxes with different dimensions were used for each probe to find the minimum measurement-chamber dimension that give us a proper lecture. Different ranges of EC (usual on goat milk) were studied according with the ranges described on [5,10–12].

Several samples, comprising saline water solutions, UHT cow milk and fresh goat milk were measured with the reference conductimeter. Then, samples were manually passed through different volume measurement chambers in order to find the minimum dimension of the needed volume.

#### 2.4.2. On-Line Testbed

##### First Evaluation of Commercial Probes

A portable milking machine and an artificial udder available at the laboratory (see Figure 4) were used for this experiment. A daily manual milking were done to several goats from a commercial farm. The fresh milk was put on separate jars and translated to the milking laboratory of the teaching farm of the Escuela Politécnica de Orihuela, Universidad Miguel Hernandez. Samples were heated to 37 °C (a similar temperature similar to the inside of the goat udder).

**Figure 9.**
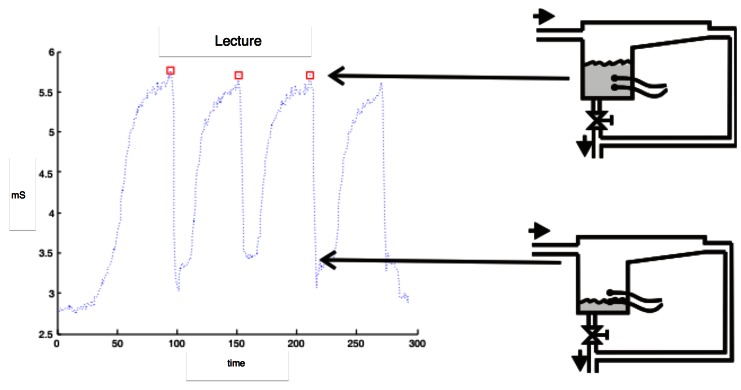
Scheme of the manual valve functioning.

Next, the temperature of each sample was checked, and the EC was obtained using the reference conductimeter, with the same temperature compensation of the checked probe (25 °C or 20 °C). After that, milk was introduced in one of the halves of the artificial udder and it was connected to the milking machine. The emission flow of the udder was regulated to 0.4 L/min. The manual valve allocated after the outlet of the measurement chamber was manually controlled in order to allow the accumulation of milk in the interior of the measurement chamber. When the level of milk was right, i.e., the probe was completely submerged in the fluid, the value of the EC was written down. Next, the valve was opened and the measurement chamber was emptied, closing it afterwards, and starting again to fill the measurement chamber with new milk. The process was repeated until the artificial udder was emptied. Process can be viewed on the scheme of Figure 9.

Ten 1 liter sample bottles were milked before cleaning processes, number similar to animals milked during a milking in a commercial farm of small ruminants. The cleaning process included the measurement chamber, the EC probe and the inlet and outlet ports, and it was similar to the conventional cleaning processes on commercial milking machines: open-loop rinsing, closed-loop washing with heated water (40 °C) and alkaline detergent, and again open-loop rinsing. For every 2 washings, a closed-loop washing with acid detergent was added before the last open-loop rinsing in order to avoid deposit of carbonates.

This methodology was repeated for each of the conductimeters 5 times (n = 5 milkings with n = 10 samples each one). On each sample, next data was obtained: deviation between data, difference between the maximum EC and reference one, number of readings with each sample of 1 liter, reading of the appearance of the maximum EC. The average values of each milking for each conductimeter were calculated from the data of the samples.

##### Evaluation of the Temperature Effect

A second experiment was done using the online laboratory setup. The experiment consisted in evaluating the effect of the temperature variations on the measurement of the probes because in previous experiment a rough effect on the lectures of the probes was observed due to the influence of the vacuum. We postulated that the vacuum generated a great variation of the temperature in the measurement chamber, due to evaporation processes. To check this effect and to study the dynamics of the commercial probes, C2 and C3 conductimeters were used (C1 did not have access to the lecture of the integrated Pt100 sensor). Five 1 liter saline-water samples with different EC were used. Each sample was milked with the same flow and vacuum parameters as milk was before. For each conductimeter, the average and standard deviation of lectures and time of acquisition and order of stable lecture were written down.

#### 2.4.3. On-Line Milking Parlour Experiment

**Figure 10.**
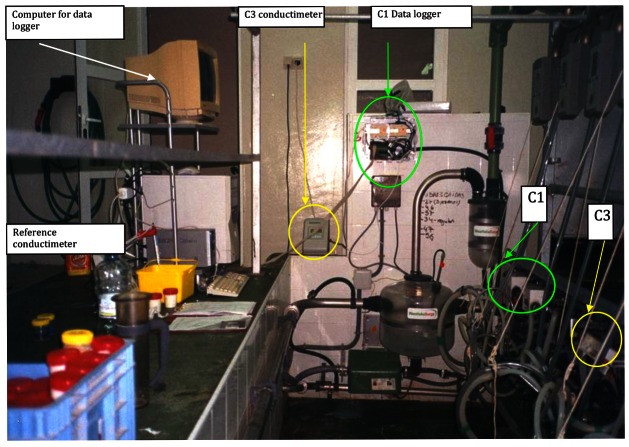
Setup of the conductimeters in the milking parlour.

After previous experiments, C1 and C3 conductimeters were selected to be integrated into a real milking parlour. The Educational and Experimental Farm of UMH was selected (see Figures 6 and 10).

Twenty four goats at their third month of lactation were selected for this experiment. Animals were homogeneously distributed into 2 groups of 12 goats (24 halves) by group and randomly assigned one conductimeter (C1 or C3) to be tested. Goats were milked daily, and gland milk EC was recorded on-line by the assigned conductimeter, during 120 days at C1 group, and 127 days at C3 group. Tested conductimeters were calibrated at the beginning of the experiment, but never again. In order to check the behaviour of the tested conductimeters, EC of a representative sample of the whole milking of every gland was analyzed using reference conductimeter (CE0) after milking, giving the result with the same compensation of temperature of the tested conductimeter (20 °C if C1 and 25 °C if C3). CE0 was recorded the next sampling days at C1 group: first 45 consecutive days, once after other 15 days, once after other 30 days, and once after other 30 days. At C3 group, CE0 was recorded the next sampling days: first 45 consecutive days, once after 7 days, once after 15 days, once after other 30 days and once after other 30 days. Reference conductimeter was calibrated every sampling day.

Due to the features of the data loggers of each conductimeter, C1 measures were taken with a frequency of 0.5 s, and C3 measures were obtained every 1 s.

C1 data was read directly over Matlab®, using the analog output of the C1 data logger and a 512PG I/O card from National Instruments.

C3 data was read using the RS232 communication port of the C3 data logger. The rough data was stored as a plain text and then imported over Excel® software. Excel® software was used to format the data to a “.csv” file format able to be read from Matlab®.

**Figure 11.**
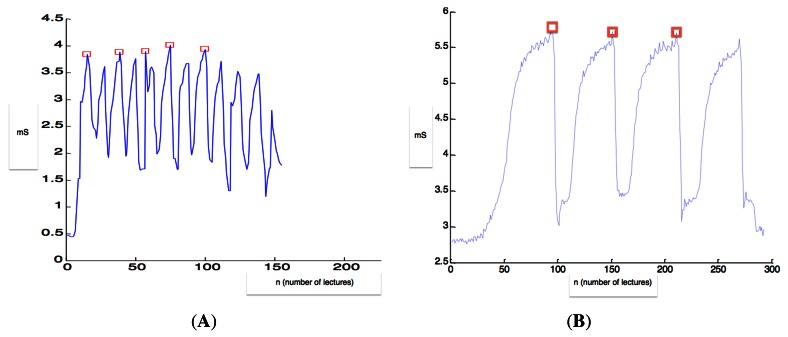
Explanation of the selection of the local maxima with Matlab® for the C1 (**A**) and C3 (**B**) probes.

Using Matlab®, a simple script for getting the EC measures from the rough data was developed. An explanation of the script can be followed on Figure 11. The maximum EC at every fulfill of the measurement chamber was stored and called “local maximum (LM)”. The algorithm of the script was similar to the one used in [4] or [3]. The average of the local maxima was calculated for every tested conductimeter. Due to the different volumes of the measurement chamber of C1 and C3 probes, in the case of C1 the average of the three maximum LM was calculated, while for C3 the average of the five maximum LM was calculated. On Figure 11 the rough data and the measures got using the Matlab® script are observed. The effect of the filling of the measurement chamber (explained on Figure 9) can be easily observed. EC2 algorithm was the maximum of the LM calculated.

#### 2.4.4. Statistical Analysis

Accuracy of tested conductimeters was analyzed for EC1 and EC2 algorithms considered, comparing with EC0 lecture (EC0-EC1 and EC0-EC2). Average differences and standard deviation of differences was calculated.

The accuracy evolution of tested conductimeters was analyzed among time. Anova analysis of accuracy of EC1 and EC2 algorithms were calculated for both tested conductimeters (Proc GLM, SAS 9.1.). The statistical model used was: *Y_ij_* = *μ* + *α_i_* + *e_ij_*. *Y_ij_* was the dependent variables (accuracy of CE1 = EC1 — EC0; accuracy of EC2 = EC2 — EC0, of every tested conductimeter: C1 and C3); *μ* = mean; *α_i_* = the effect of the period of measurement (C1 analysis included 6 levels: the first 45 days of the experiment were divided into 3 levels of 15 days each, 3 levels corresponding to the last measurements in isolated days; C3 analysis included the same first 3 levels and other 4 levels more corresponding to the last measurements carried out in isolated days); e*_ij_* = the residual error.

## 3. Results and Discussion

### 3.1. Off-Line Measurement Experiment

The results of this experiment allowed to build the boxes for each one of the probes. Figure 8 shows the boxes developed for C1, C2 and C3. The inner dimensions of the measurement chamber for each probe are detailed on Table 2. Developed boxes were employed in the experiments of on-line testbed and on-line milking parlour.

### 3.2. On-Line Testbed Experiment

#### 3.2.1. First Evaluation of Commercial Probes

In order to study the evolution of EC among milkings, for every testbed conductimeter we calculated the difference between the maximum EC read by the commercial probe and the reference EC, the number of measurements of a milking, and the order of the lecture where the maximum EC was obtained (see Table 3).

**Figure 12.**
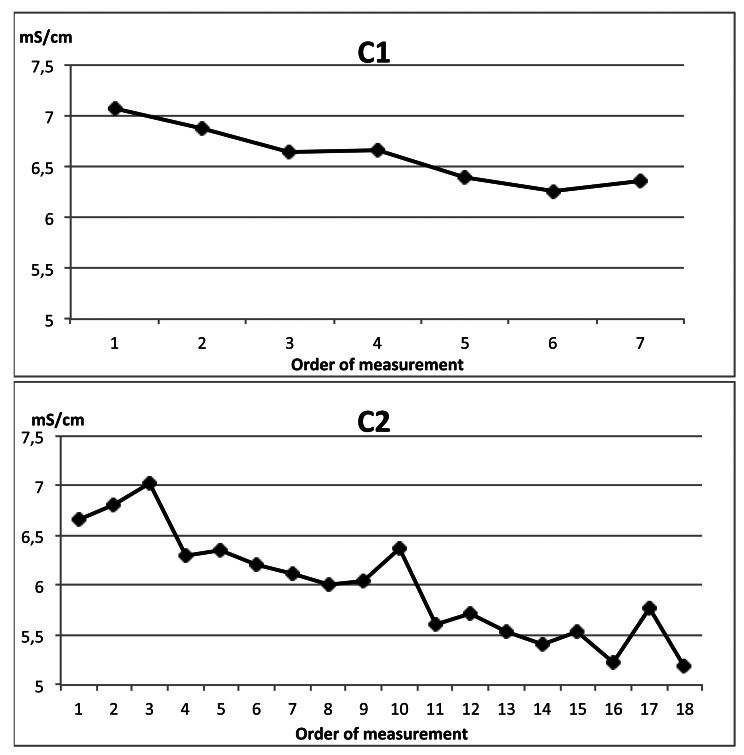

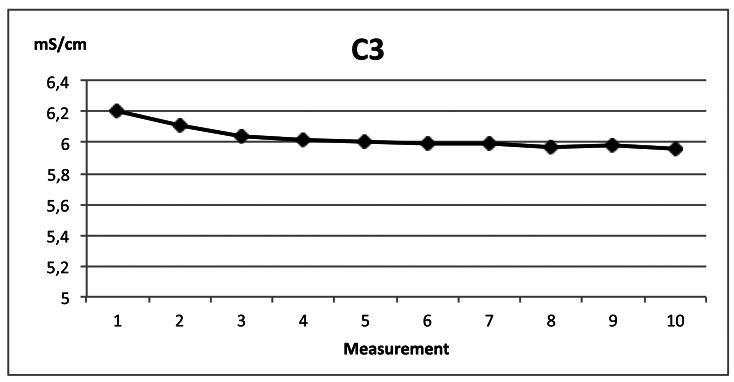
Examples of the evolution of EC measures during one milking.

Table 3 shows that the conductimeter which offered more stability and thus a lower average of standard deviation of the measures was the C3 (0.06 mS/cm) following the C1 (0.32 mS/cm), being C2 the probe that presented a larger standard deviation average and thus, a lower repeatability of the readings (0.52 mS/cm). In Figure 12 some examples of the evolution of the measures are shown: C3 presented a more stable lecture of the EC, while C1 and C2 presented some variations due to the parameters of the milking process, as the sample is homogeneous during the milking.

**Figure 13.**
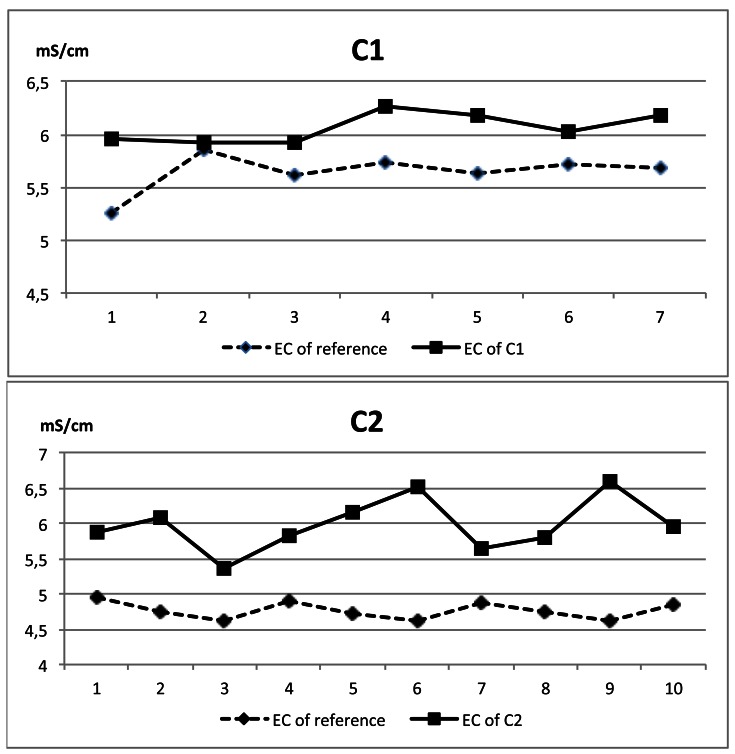

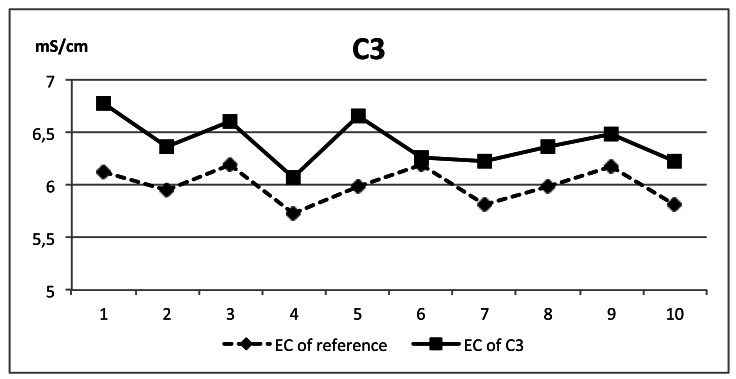
Examples of the performance of commercial probes versus laboratory probe on samples measured consecutively.

Regarding to the difference between the maximum values of EC and reference EC, the C3 probe showed the smallest difference in absolute values (0.33 mS/cm), similar to C1 (0.34 mS/cm), and C2 showed larger differences (2.46 mS/cm). This parameter indicates the goodness of the calibration of the probes in comparison with the reference probe. In Figure 13 some examples of the performance of the commercial probes tested with 10 different samples are shown.

Regarding the number of lectures that could be done for 1 liter of sample, C2 achieves a greater number of lectures (15.34) followed by C3 (11.75) and C1 (5.58). This is obvious due to the dimensions of the measurement boxes. It is important due to the nature of the algorithm for the analysis of rough data. As it was shown on Figure 11, the algorithm is dependent of the number of local maxima, which is the number of achieved lectures. So, as much lectures the system is able to do, the better on line measurement of the real EC.

#### 3.2.2. Evaluation of the Temperature Effect

As it can be seen in Table 4, C3 system presented a minor deviation in the temperature lectures (1.33 *vs.* 2.15 °C) together with a faster establishment of the steady state (43 s *vs.* 106 s) which indicates a better dynamic response for working in discontinuous vacuum processes than C1. This feature has a very important influence on the EC measurement, due to the temperature compensation process of the commercial systems. A wrong lecture of temperature will produce a EC error that would avoid its use as a mastitis detection system.

Regarding the values of Table 5 the same pattern for the evolution of the EC was observed (obvious due to the relation mentioned before). The C2 system had a higher time for steady state than the C3. This could be because of the geometry of the probes. C3 probe is capsuled on crystal while C2 probe on inox steel. The different heat transmission coefficients of materials could be the reason of the differences.

**Figure 14.**
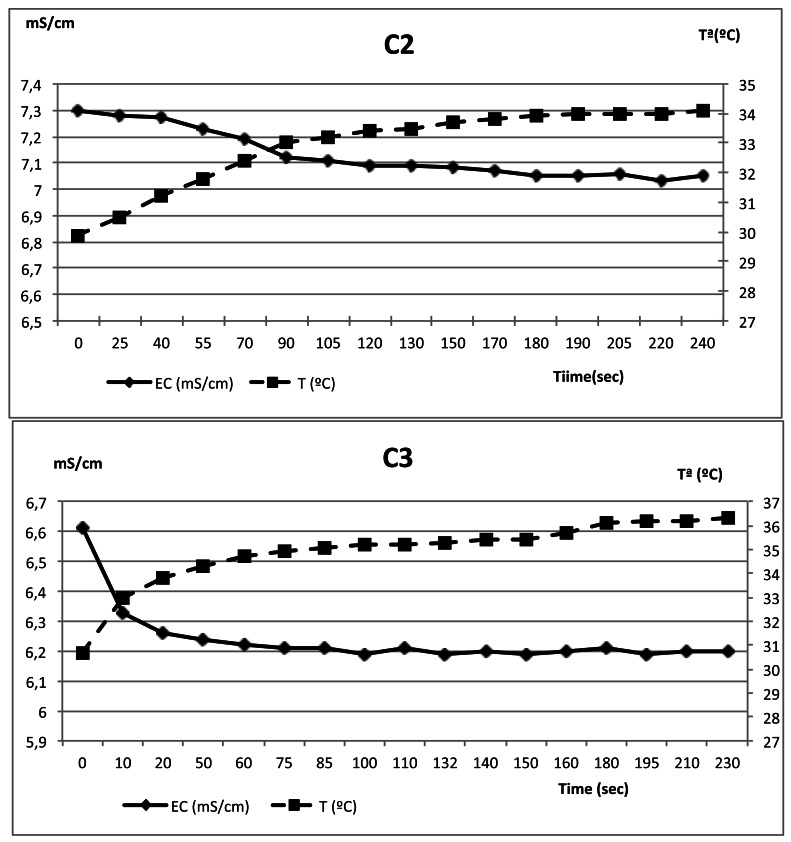
Evolution of lectures of temperature and EC in saline water solutions.

Figure 14 shows an example of the evolution of the EC and temperature during the milking of saline sample on both equipments. It is important to note that the effect of wrong temperature acquisition could have an effect of more than 0.5 mS, which is incompatible with the use of EC for mastitis detection. A probe with less time for steady state is desirable if we want to use it for online mastitis detection.

Considering all the results obtained during this testbed experiment, the research team decided to select C1 and C3 for the milking parlour experiment.

### 3.3. On-Line Milking Parlour Experiment

Table 6 shows the results of the first evaluation of C1 and C3 systems in a milking parlour. EC values agree to observed by Park [9], Schuppel and Schwope [8] and Diaz *et al.* [11,12] at different goat breeds.

Both developed algorithms to be used by tested conductimeters (EC1 and EC2) presented on-line measures higher than the reference value (CE0). C1 showed values closer to CE0 than C3, indicating a higher accuracy. EC1 algorithm presented a higher accuracy than EC2 for both tested conductimeters. The standard deviation of EC1 (average of local maxima) and EC2 (maximum of local maxima) of C1 were similar and higher than EC0 (reference conductimeter) (0.38, 0.7 and 0.46 mS/cm, respectively). C3 conductimeter presented higher standard deviation than C1 and EC0.

EC1 algorithm had higher accuracy than EC2 for both conductimeters (Table 7) probably because EC1 is the average of several measurements during milking and EC0 was measured in a representative sample of the whole milking, and EC2 was the higher local maxima registered, and obviously higher than EC1. The C1 conductimeter showed lower difference between EC1 and EC2 algorithms (0.1 mS/cm) than C3 (0.23 mS/cm) because C3 registered higher number of measurements at every sample due to the measurement chamber had lower volume, causing a higher dispersion of local maxima values.

Both conductimeters suffered an undesirable variation of accuracy among the on-line milking parlour experiment (Tables 8 and 9). C1 reduced the difference with EC0 and, opposite to it C3 increased the difference among time. This uncalibration process questions the proper performance of conductimeters for goats mastitis detection (the application objective) because the expected effect of mastitis on milk EC can be masked by the uncalibration observed.

## 4. Conclusions

An on-line and quick measurement of the temperature is needed to obtain proper EC data for mastitis detection. The special conditions of the milking, with vacuum pulsation and the evaporation due to the vacuum, impose the need of maintaining the probe under immersion continuously, something that was not achieved with the prototypes presented in this paper.

At tested experiments, the better global performance was exhibited by the C3 probe, that had less variation of the lectures and a faster stabilization of the measures. The worst performance was exhibited by the C2 probe that suffered a degradation of the lectures during the milking, maybe due to the deposition of fat on the surface of the probe.

C3 and C1 were employed at on-line milking parlour experiment. The study showed that the on-line values obtained from goat halves were similar to the off-line values reported on the literature. Nevertheless, the dynamics of the lectures of C1 (slow answer to temperature variations) avoid its use of this prototypes as a solely sensor for mastitis detection. Other consideration is about the dimension of the probe. The current dimensions are too big to the expected milk production of a single goat gland, and it reduces the number of lectures taken during the milking. The high correlation with the computation criteria selected (EC1 or EC2) in both probes indicated that trying to maximize the number of lectures is also a requirement (minimize the size) for a better probe design.

So, although the C3 probe presented the best performance to be installed in a milking parlour, it did not give the needed performance to be used as a solely method for on-line mastitis detection. Nevertheless, this study allowed authors to find the requirements for a new probe that minimize the detected problems. This constraints are briefly presented.

### 4.1. Requirements for a New Probe

A new probe design is needed for its use in milking parlours of small ruminants. The design constraints are:

- Small size. Minimizing the size we will be able to have as much number of lectures as possible of every sample.
- Measurement range. The values for Murciano-Granadina breed varies between 4.75 and 6.6 mS/cm, although these values can be elevated by the presence of mastitis.
- To integrate a temperature sensor with fast dynamics.
- The probe must be under immersion continuously. This constraint implies that the measurement chamber should be always full of milk, although this milk should also be recirculated in order not to measure the same sample all the time.
- Auto-calibration of the conductivity meter is needed. The several factors that affect the measurement of the EC and the small differences between a healthy gland and infected halves impose that the conductivity meter should be easy and frequently calibrated.

A conductimeter with this requirements has been presented by the authors under the patent ES U201100694.

## Figures and Tables

**Table 1.** Dimensions of tested probes. Source: own elaboration.

**Probe**	**Total length**	**Diameter**	**minimum submerged length**
C1	94 mm	31 mm	42 mm
C2	130 mm	12 mm	25 mm
C3	130 mm	12 mm	35 mm

**Table 2.** Minimum dimension of the measurement chambers.

**Probe**	**Length × Diameter × Submerged Length**
C1	80 mm × 50 mm × 45 mm
C2	45 mm × 25 mm × 25 mm
C3	45 mm × 25 mm × 25 mm

**Table 3.** Electrical conductivity measurements, deviations from reference, number of lectures during milking, and order of the maximum EC lecture, of the tested conductimeters at on-line testbed experiment.

**Testbed conductimeter**	**Overall EC results**				**DEC**		**Number of EC lectures at one milking**		**Order of lecture of max EC at one milking**	

	**Number of EC lectures**	**Average Average EC(mS/cm)**	**SD (mS/cm)**	**Average VC%**	**Average (mS/cm)**	**SD (mS/cm)**	**Average**	**SD**	**Average**	**SD**
C1	255	6.07	0.32	5.22	−0.34	0.44	5.58	0.67	1.58	0.82
C2	767	6.32	0.52	8.36	−2.46	0.55	15.34	1.73	2.12	1.94
C3	564	6.17	0.06	1.05	−0.33	0.18	11.75	0.47	2.23	1.58

EC: electrical conductivity; SD: standard deviation, VC: variation coefficient; DEC: reference EC-maximum EC registered by tested conductimeters at every sample; 50 samples were milked by every tested conductimeter;

* The average of the SD (and VC) of lectures obtained at every sample analysis was calculated.

**Table 4.** Temperature dynamics in C2 and C3 probes at on-line testbed experiment.

**Probe-sample**	**Temperature evolution**			

	**Mean (°C)**	**Std.dev. (°C)**	**steady state time (s)**	**N° of lectures**
C2-1	32.53	3.69	120	12
C2-2	32.48	0.93	100	16
C2-3	32.90	1.35	90	16
C2-4	31.20	2.52	100	11
C2-5	31.32	2.27	120	16
Average	32.08	2.15	106	14.2
C3-1	35.38	1.93	28	15
C3-2	34.47	1.45	40	17
C3-3	34.91	1.40	60	17
C3-4	34.61	1.42	62	16
C3-5	31.76	0.44	25	17
Average	34.23	1.33	43	14.4

**Table 5.** EC dynamics in C2 and C3 probes at on-line testbed experiment.

**Probe-sample**	**EC evolution**			

	**Mean (mS/cm)**	**Std.dev. (mS/cm)**	**steady state time (s)**	**N° of lectures**
C2-1	9.55	0.32	120	12
C2-2	5.11	0.14	85	16
C2-3	7.13	0.09	90	16
C2-4	5.31	0.20	100	11
C2-5	6.58	0.16	120	16

Average	6.74	0.18	103	14.20

C3-1	5.92	0.06	15	15
C3-2	7.46	0.06	40	17
C3-3	6.24	0.10	20	17
C3-4	8.27	0.07	30	16
C3-5	6.82	0.09	25	17

Average	6.94	0.08	26	14.40

**Table 6.** Electrical conductivity (EC, mS/cm) of samples analyzed during on-line milking parlour experiment.

	**Reference EC**				**EC1**				**EC2**			

	**N**	**Average**	**SD**	**VC**	**N**	**Average**	**SD**	**VC**	**N**	**Average**	**SD**	**VC**
C1	1,110	5.31	0.46	8.61	1,110	5.62	0.38	6.83	1,110	5.72	0.37	6.52
C3	987	5.73	0.45	7.97	987	6.17	0.59	9.51	987	6.40	0.58	9.01

EC1: See Material and Methods chapter for definition of EC1 and EC2; N: Number of observations considered; SD: standard deviation (mS/cm); VC: variation coefficient (%).

**Table 7.** Accuracy of tested conductimeters during on-line milking parlour experiment.

	**Accuracy EC1**			**Accuracy EC2**		

	**N**	**Average**	**SD**	**N**	**Average**	**SD**
C1	1,110	−0.31	0.35	1,110	−0.40	0.35
C3	987	−0.44	0.45	987	−0.67	0.38

Accuracy: EC reference (CE0)—EC algorithm tested (EC1 or EC2); N: Number of observations considered; SD: standard deviation (mS/cm).

**Table 8.** Results of ANOVA analysis of C1 conductimeter accuracy (mS/cm) among time of on-line milking parlour experiment.

**Accuracy** 1	**Statistic**	**Days of experiment**					

		**1–15**	**16–30**	**31–45**	**60**	**90**	**120**
EC1	Mean	−0.425^a^	−0.361 b	−0.225 c	0.281 d	0.065 e	−0.062 e
	SE 2	0.017	0.0175	0.017	0.066	0.068	0.071

EC2	Mean	−0.511^a^	−0.455 b	−0.321	0.198 d	0.060 e	−0.229 e
	SE 2	0.017	0.017	c 0.017	0.066	0.068	0.071

	N	345	346	351	24	23	21

a–e different scripts within a row indicate significant difference (*p* < 0.05);

1 Accuracy: Difference of reference electrical conductivity (EC0) and algorithms considered (EC1 or EC2; See EC1 and EC2 definitions at Material and Methods chapter;

2 SE: standard error; N: Number of observations.

**Table 9.** Results of ANOVA analysis of C3 conductimeter accuracy (mS/cm) among time of on-line milking parlour experiment.

**Accuracy** 1	**Statistic**	**Days of experiment**						

		**1–15**	**16–30**	**31–45**	**60**	**75**	**105**	**135**
EC1	Mean	−0.285 a	−0.559 b	−0.472 c	−0.480 bc	−0.365 abc	−0.408 abc	−0.285 ac
	SE 2	0.025	0.025	0.026	0.096	0.097	0.117	0.109

EC2	Mean	−0.493 a	−0.793 b	−0.714 c	−0.772 bc	−0.605 ac	−0.639 abc	−0.675 abc
	SE 2	0.021	0.021	0.020	0.081	0.082	0.099	0.093

	N	302	304	275	21	20	14	16

a–c different scripts within a row indicate significant difference (*p* < 0.05);

1 Accuracy: Difference of reference electrical conductivity (EC0) and algorithms considered (EC1 or EC2); See EC1 and EC2 definitions at Material and Methods chapter;

2 SE: standard error; N: Number of observations.
